# Coordinated expression of vascular endothelial growth factor A and urokinase-type plasminogen activator contributes to classical swine fever virus Shimen infection in macrophages

**DOI:** 10.1186/s12917-019-1826-8

**Published:** 2019-03-08

**Authors:** Xiaocheng Gong, Aoxue Hu, Xuepeng Li, Jun He, Zhongxing Wu, Xi Zuo, Pengbo Ning

**Affiliations:** 1 School of Life Science and Technology, Xidian University, Xi’an, Shaanxi 710071 People’s Republic of China; 2 Engineering Research Center of Molecular and Neuro Imaging Ministry of Education, Xi’an, Shaanxi 710071 People’s Republic of China

**Keywords:** CSFV Shimen, VEGFA, uPA, Macrophages

## Abstract

**Background:**

The Shimen strain of classical swine fever (CSF) virus (CSFV) causes CSF, which is mainly characterised by disseminated intravascular haemorrhage. Macrophages are an essential component of innate immunity against pathogenic microorganisms; however, the role of macrophages in CSF pathogenesis remains unclear. To illuminate the infective mechanism of CSFV, we used gene co-expression networks derived from macrophages infected with CSFV Shimen and CSFV C as well as uninfected macrophages to screen key regulatory genes, and their contributions to the pathogenesis of CSF were discussed.

**Results:**

Vascular endothelial growth factor A (*VEGFA*) and plasminogen activator, urokinase (*PLAU*, which encodes urokinase-type plasminogen activator [uPA]) were identified as coordinated genes expressed in macrophages by gene co-expression networks. Quantitative polymerase chain reaction and western blot analysis confirmed that *VEGFA* and *PLAU* were significantly up-regulated at both the transcription and translation levels after infection. Further, confocal microscopy analysis proposed that the VEGFA and uPA proteins were temporally co-localised with the CSFV protein E2.

**Conclusions:**

Our findings suggest that co-expression of *VEGFA* and *PLAU* in macrophages contributes to CSFV Shimen infection and serves as a significant avenue for the strain to form an inflammatory microenvironment, providing new insight into the mechanisms of CSF caused by a virulent strain.

**Electronic supplementary material:**

The online version of this article (10.1186/s12917-019-1826-8) contains supplementary material, which is available to authorized users.

## Background

The Shimen strain of classical swine fever virus (CSFV) causes an especially infectious disease in domestic pigs known as classical swine fever (CSF), which has been listed as a highly contagious disease by the World Organisation for Animal Health [1]. Acute CSF is typically accompanied by haemorrhagic lymphadenitis and diffuse haemorrhage in the skin, kidney, and other organs, often resulting in high mortality within a short period of time. The mechanism by which diffuse haemorrhage occurs in CSF is not yet fully understood.

Vascular endothelial growth factor A (VEGFA), the prototypical member of the VEGF family, plays important roles in mammalian vascular development and in diseases relating to abnormal growth of blood vessels. Tumours and inflammatory disorders often trigger pathological angiogenesis to generate a new vascular supply; detailed pathological studies have revealed the VEGFs as active participants in these processes [2]. Currently, it is accepted that VEGFs produced in tumour cells auto-stimulate neo-angiogenesis of neoplastic tissue and tumour growth [3]. Meanwhile, the role of VEGFA in different inflammatory diseases has been explored, and the blockade of its signalling is considered a protective strategy in the treatment of such diseases [4]. In recent years, increased levels of VEGFA have been observed in pro-inflammatory environments created by viral infections in humans [5, 6], further motivating study of VEGFA.

*PLAU* encodes urokinase-type plasminogen activator (uPA). Unlike VEGF, uPA promotes vascular permeability and angiogenesis through proteolytic degradation of the extracellular matrix, which assists tumour invasion and metastasis [7]. In the 1970s, uPA was reportedly up-regulated in Rous sarcoma virus-transformed chicken cells [8]. Later, *PLAU* was associated with the complex phenotype of human cancer, and high serum levels of uPA have been associated with worse overall survival rates among patients with cancer [9]. However, relatively little information is available about the role of uPA in virus–host interactions.

Macrophages are an essential component of innate immunity, with multiple functions in both inhibition and promotion of cell proliferation as well as tissue repair [10]. Despite causing acute organic damage, the CSFV Shimen strain causes no apparent cytopathic effect, but rather propagates efficiently in macrophages [11]. Whether the macrophage-mediated inflammatory response promotes the haemorrhagic mechanism of CSF is unclear. Based on analysis of a digital gene expression (DGE) profile obtained previously [11], the present study identified VEGFA and uPA as potential pathogenic factors co-expressed in CSFV Shimen-infected macrophages. The different effects of CSFV Shimen and CSFV C infection on VEGFA and uPA expression were detected. CSFV C can complete its infection cycle without any pathological symptoms [12], and it was as the control to help understand the contribution of CSFV Shimen to pathogenesis of CSF.

## Methods

### Experimental design

DGE analysis [13] performed on CSFV Shimen-, CSFV C-, and mock-infected macrophages has been well-described in our previous report [11]. In the present study, series cluster analysis was applied to identify significantly up- and down-regulated genes in CSFV Shimen vs CSFV C and control groups by Fisher’s exact and multiple comparison tests [14]. Further, the co-expression (Pearson correlation coefficient) of VEGFA and PLAU was calculated by Java code [15], and gene co-expression network analyses were carried out to track the interactions among the up- and down-regulated genes. Pearson correlation coefficients were compared for each pair of genes, and the significantly correlated pairs were used to construct a network [16] in which “key regulatory genes” (*P*-Value < 0.05) were identified to elucidate the role of macrophage status in intrinsic susceptibility to CSFV Shimen infection. Protein-protein interactions involving VEGFA were analysed using STRING (version 10.5; https://string-db.org/cgi/input.pl?sessionId=Ym91vu6hSHFN&input_page_show_search=on). Several proteins interacting with VEGFA were selected from the STRING database for *Sus scrofa*, one of the ELIXIR Core Data Resources. GO analysis of the proteins was carried out using the GO Term Enrichment tool in AmiGO (http://amigo1.geneontology.org/cgi-bin/amigo/term_enrichment?session_id=), the bar chart for which was constructed by OriginPro 2016 (OriginLab Corp., Northampton, MA, USA). Further tests were conducted to confirm whether the “key regulatory genes” responded to CSFV Shimen infection by inoculating the virus at an MOI (multiplicity of infection) of 5 in macrophages for 0, 12, 24, and 48 h, according to previously described methods [11].

### Quantification of mRNA expression using quantitative polymerase chain reaction (qPCR)

Relative mRNA expression was determined by quantitative PCR. Specific oligonucleotide primers for each gene were as follows: *VEGFA* (5′-CCTTGCTGCTCTACCTCCAC-3′ and 5′- CACTCCAGACCTTCGTCGTT-3′) and *PLAU* (5′-CGCAAGCTGTGAAATCGTC-3′ and 5′- TTCGCTGCCGTAGTAATGG-3′). qPCR analysis of each gene was performed in triplicate, and the 2^-ΔΔCt^ method was applied to calculate the relative expression levels.

### Western blot analysis

The macrophages were lysed with RAPI buffer (Beyotime Institute of Biotechnology, Shanghai, China) and used for western blotting as previously described [11]. Primary antibodies against VEGFA (Ominimabs, Alhambra, CA, USA), uPA (Santa Cruz Biotechnology, Dallas, TX, USA), E2 (MssBio, Guangzhou, China), and β-actin (Biodragon Immunotechnologies, Beijing, China) were used in this study. β-Actin was used as a common internal control to normalise the relative transcription and translation expression of each gene.

### Confocal microscopy

CSFV- or mock-infected macrophages were washed in phosphate buffered saline (PBS) and fixed with methanol/acetone (1:1) for 20 min at 25 °C ± 2 °C followed by a 10-min permeabilisation with 1% Triton X-100 in PBS. After three washes in PBS, the samples were incubated with mouse anti-E2 antibody and rabbit anti-VEGFA antibody or uPA antibody for 1 h at 25 °C ± 2 °C, followed by staining with donkey anti-rabbit IgG conjugated to Alexa Fluor® 594 and donkey anti-mouse IgG conjugated to Alexa Fluor® 488 (Thermo Fisher Scientific, Waltham, MA, USA) at a 1:200 dilution for 1 h at 25 °C ± 2 °C. The nuclei in macrophages were stained with 4′,6-diamidino-2-phenylindole (DAPI). Confocal images were obtained with a laser-scanning confocal microscope (LSM 510 META; Carl Zeiss, Oberköchen, Germany).

### Statistical analysis

Statistical analyses were conducted by one-way ANOVA using SPSS 16.0 (SPSS, Chicago, IL, USA), and differences with a *p*-value < 0.05 were considered statistically significant.

## Results

### *VEGFA* and *PLAU* expression are positively correlated with CSFV Shimen infection

**Fig. 1:**
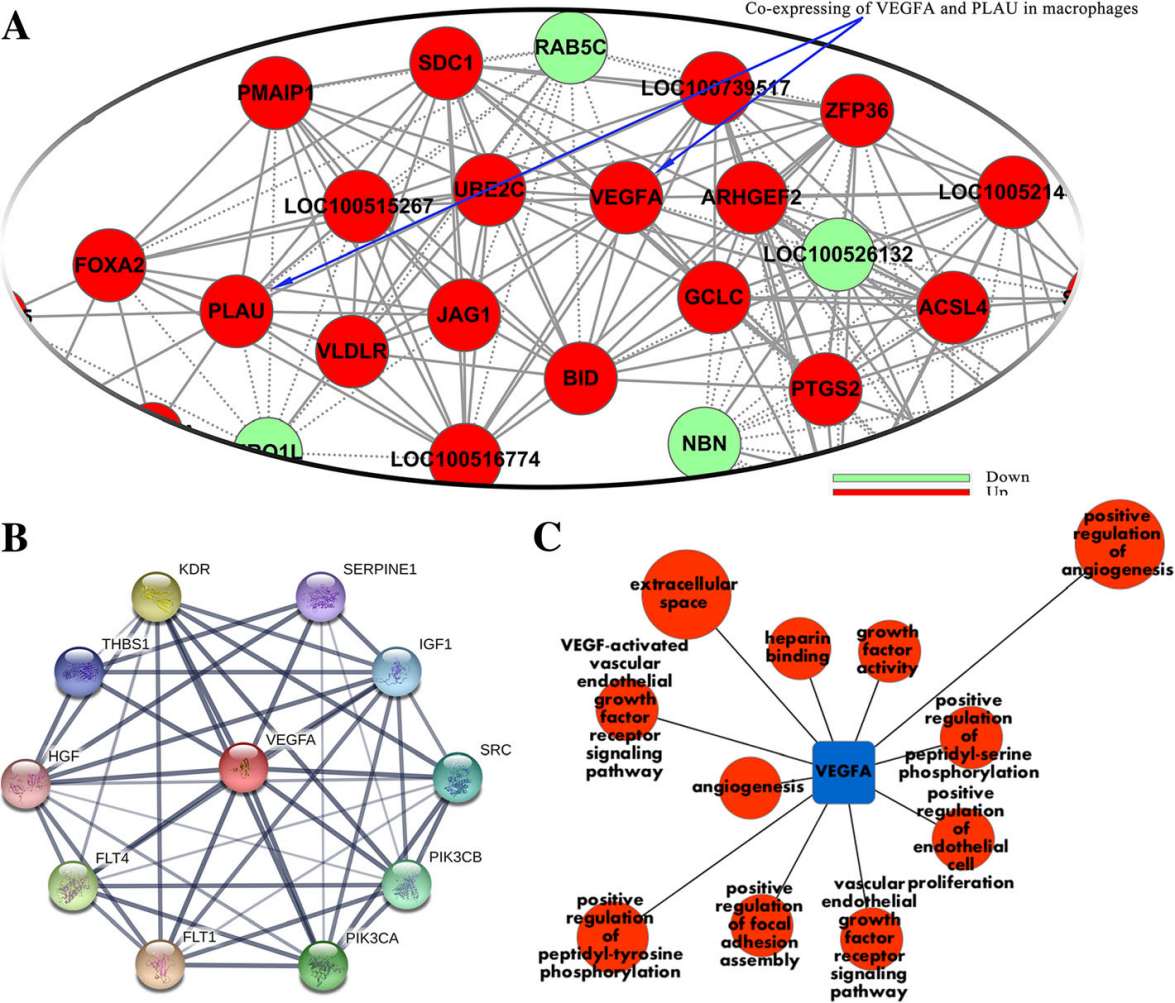
The role of *VEGFA* and *PLAU* in the co-expression network of cells infected by CSFV Shimen. (**a**) The co-expression network shows that *VEGFA* and *PLAU* are up-regulated in CSFV Shimen-infected cells compared to their levels in CSFV C-infected and mock-infected cells. (**b**) protein–protein interaction analysis of VEGFA. Each network node represents all proteins produced by a single protein-coding gene locus, and the edges represent protein-protein associations, which means proteins together contribute to shared functions. The meaning of the network edge is confidence, and the thickness of the line indicates the strength of data supports, which edge confidence is divided into 3 levels: medium (0.400), high (0.700), and highest (0.900). (**c**) GO analysis shows that aberrant expression of VEGFA is related to anomalous regulation of gene function groups

To understand the molecular changes caused by CSFV Shimen, we used significantly up- and down-regulated genes from the DGE database to construct a co-expression network of the CSFV Shimen vs CSFV C and control groups, and a network diagram shows this in detail (see Additional file 1). The datasets used to generate the co-expression network are included in Additional file 2. In Fig. 1a, it is clear that *VEGFA* and *PLAU* appear coordinated and up-regulated in macrophages infected with CSFV Shimen. Protein–protein interaction analysis (Fig. 1b) revealed that VEGFA interacts with several proteins, including plasminogen activator inhibitor-1 (SERPINE1), thrombospondin 1 (THBS1), and hepatocyte growth factor (HGF). GO analysis showed that anomalous expression of VEGFA is related to aberrant gene function regulation groups, such as angiogenesis and positive regulation of focal adhesion assembly (Fig. 1c).

### Up-regulation of *VEGFA* and *PLAU* by CSFV Shimen infection in macrophages

**Fig. 2:**
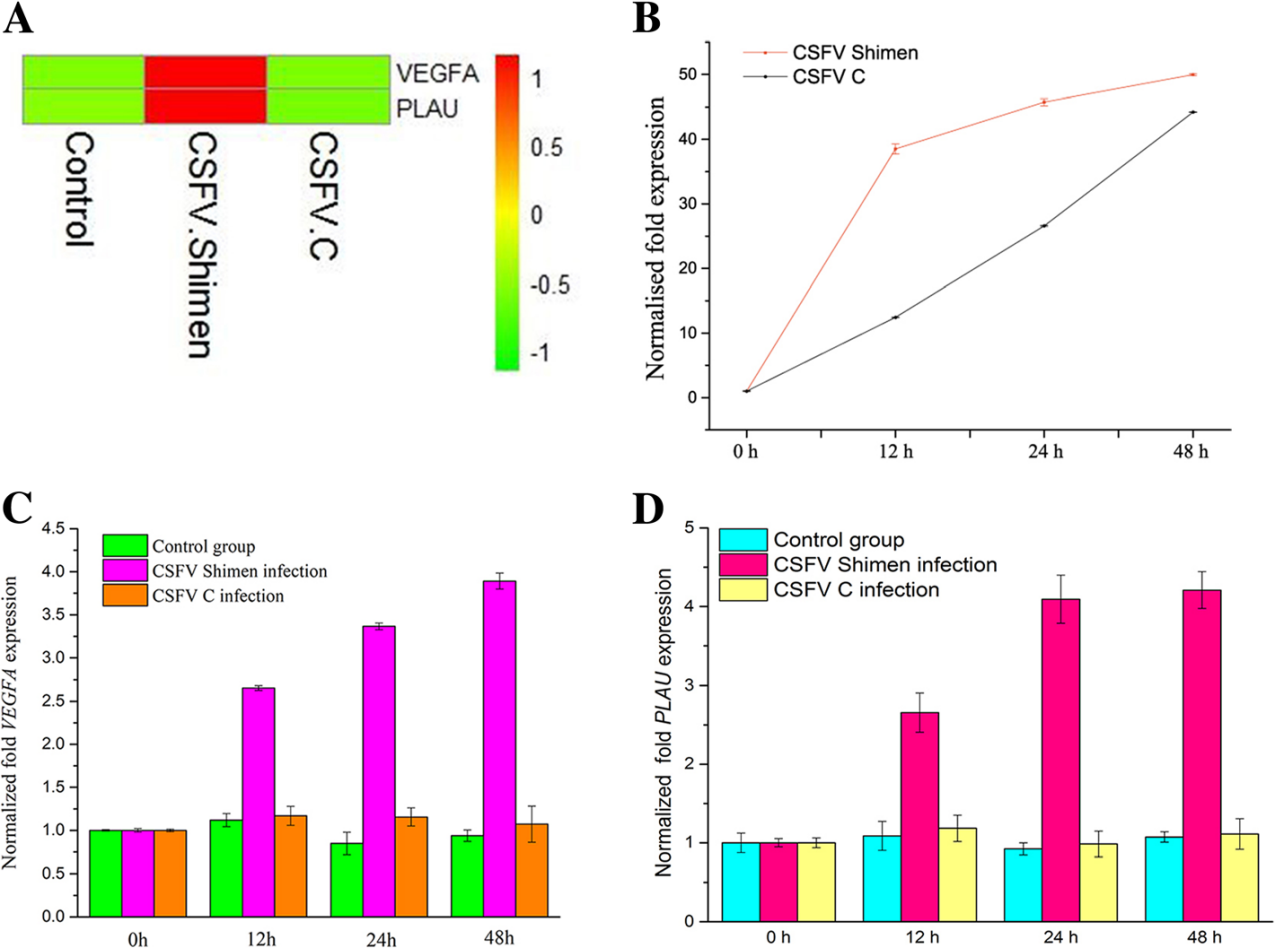
*VEGFA* and *PLAU* mRNA expression are positively correlated with CSFV Shimen infection. (**a**) DGE analysis shows that *VEGFA* and *PLAU* are up-regulated in CSFV Shimen-infected cells compared with levels in CSFV C-infected and mock-infected cells. (**b**) qPCR analysis of CSFV Shimen and C strain proliferation in macrophages. (**c**) qPCR analysis of *VEGFA* mRNA expression. (**d**) qPCR analysis of *PLAU* mRNA expression. Significant up-regulated (*p*-value < 0.05) on VEGFA and PLAU were observed between the 0 and 48 h groups for CSFV Shimen infection in qPCR analysis. Three independent qPCR experiments always obtained consistent conclusion, and one of the results was shown

As shown in Fig. 2a, DGE analysis showed *VEGFA* and *PLAU* to be significantly up-regulated in CSFV Shimen-infected macrophages compared to those in CSFV C-infected and control macrophages. qPCR confirmed infection with either CSFV Shimen or CSFV C in a time-dependent manner [11] (Fig. 2b). *VEGFA* and *PLAU* mRNA expression were measured by qPCR throughout the 48 h course of CSFV infection, and significant differences (*p*-value < 0.05) were observed between the 0 and 48 h groups for CSFV Shimen infection, suggesting a *VEGFA* and *PLAU* expression pattern similar to that predicted by DGE analysis (Fig. 2c, d).

### CSFV Shimen infection induces VEGFA and uPA expression

**Fig. 3:**
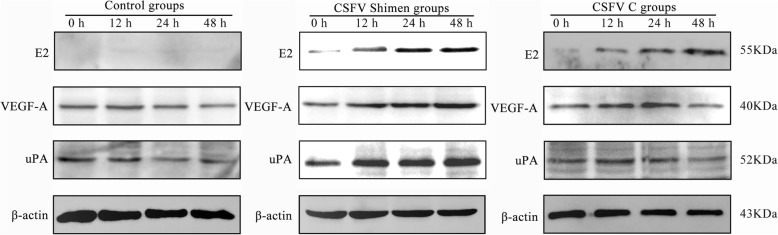
Western-blotting analysis of VEGFA and uPA in CSFV Shimen-, CSFV C-, and mock-infected macrophages. β-actin was used as a loading control

Western blot analysis confirmed the increased expression of VEGFA and uPA in macrophages after CSFV Shimen infection. E2, a structural protein in CSFV, indicated the extent of CSFV replication in cell samples. VEGFA and uPA expression in macrophages was induced in a time-dependent manner upon treatment with CSFV Shimen, which was concurrent with the increased expression of E2 48 h after CSFV Shimen infection (Fig. 3).

### VEGFA and uPA temporally colocalise with CSFV Shimen E2

**Fig. 4:**
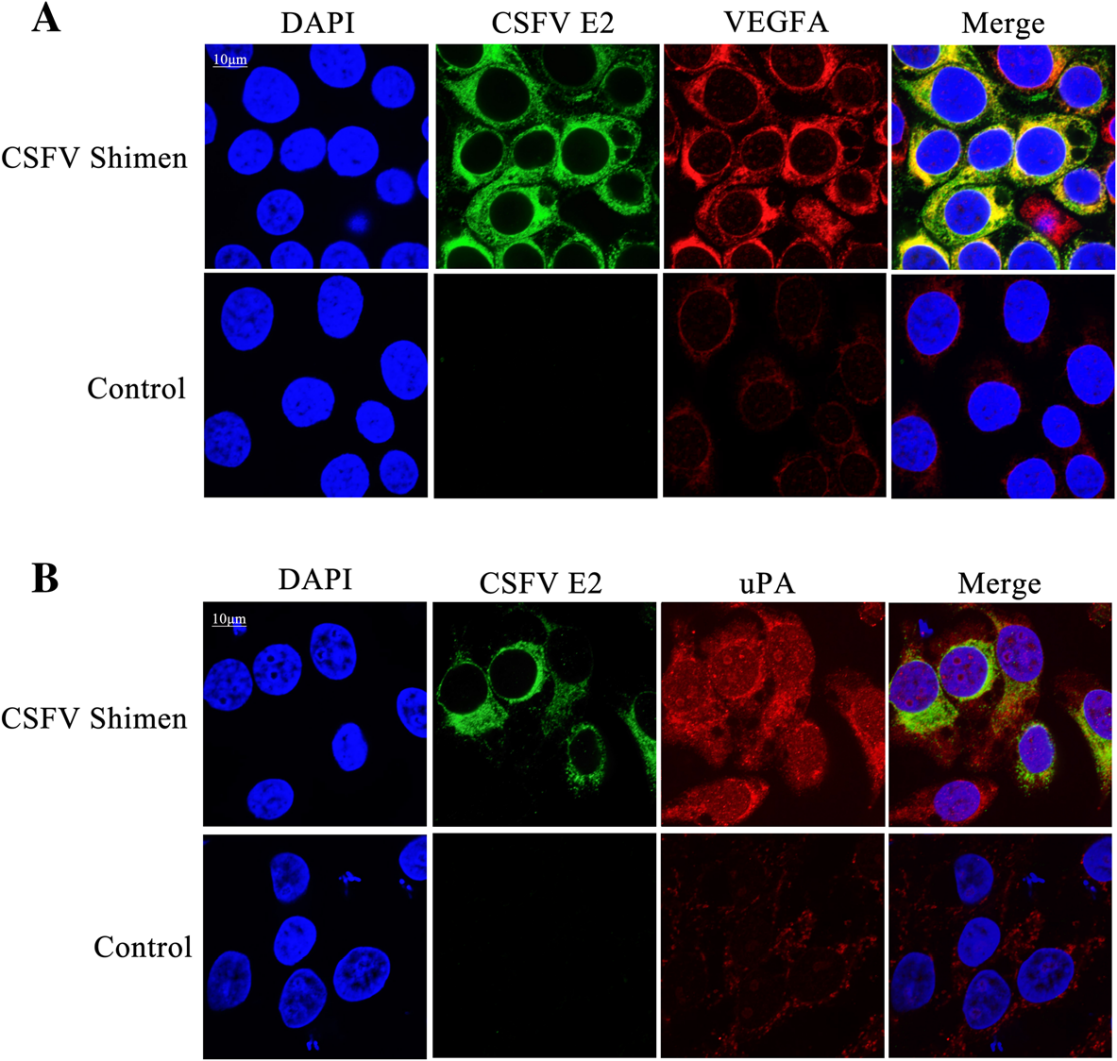
Temporal colocalisation of CSFV E2 with VEGFA and uPA in CSFV Shimen-infected macrophages. (**a**) Co-localization of CSFV E2 protein with VEGFA in macrophages infected with CSFV Shimen. (**b**) Co-localization of CSFV E2 protein with uPA in macrophages infected with CSFV Shimen. CSFV E2 antibody fluorescent signals are shown in green, VEGFA and uPA antibody signals in red, and nuclear signals in blue

Confocal microscopy was used to assess whether VEGFA, uPA, and the viral protein E2 were temporally and/or spatially co-localised in the cultured macrophages. Macrophages were treated with fluorescent labels targeting CSFV Shimen, E2, and VEGFA or uPA. Cellular localisation was tracked 24 h after CSFV Shimen infection. Temporal co-localisation of E2 and VEGFA was observed in the CSFV Shimen-infected cells, and the abundance of VEGFA in macrophages was significantly enhanced during the course of CSFV Shimen infection compared to that in uninfected controls (Fig. 4). Similar results were observed for E2 and uPA (Fig. 4). These findings suggest that VEGFA and uPA act as inflammatory cytokines in the invasion of CSFV Shimen into macrophages.

## Discussion

As a virulent strain, CSFV Shimen typically causes diffuse haemorrhagic symptoms in CSF; this is different from the CSFV C strain, which can infect pigs and produce no pathological symptoms [17]. Comparative analysis of molecular changes caused by the two strains in host cells could contribute significantly to the understanding of the related pathological mechanisms. Based on our results, we propose that coordinated expression of *VEGFA* and *PLAU* plays an important role in the inflammatory response of macrophages to CSFV Shimen infection.

This study indicated that CSFV Shimen infection triggered VEGFA responses in macrophages at both the transcription and translation levels. VEGFA recruitment by macrophages plays a crucial role in inducing inflammatory neovascularisation for pathological haemangiogenesis and lymphangiogenesis [18]. Assuming a link between angiogenesis and inflammation, the role of VEGFA in viral infectious diseases, especially in haemorrhagic lesions, has been explored. A previous report by Jones et al. revealed that varicella zoster viral infection leads to significantly increased VEGFA levels in cerebrovascular cells [5]. Herpes simplex virus 1 directly induced vascularisation of the cornea by up-regulation of VEGFA expression [19], and the VEGF trap, as an angiogenesis inhibitor, represented a functional approach to mitigating corneal neovascularisation and controlling ocular lesions [20]. A common feature of viral haemorrhagic fevers is viral entry into macrophages and dendritic cells and consequent cytopathic effects. Clinical symptoms of Crimean-Congo haemorrhagic fever (CCHF) include acute viral fever, ecchymosis, and thrombocytopenia, and a recent study showed significantly increased VEGFA levels in patients with CCHF [21]. The acute form of CSF is characterised by high fever and skin haemorrhages approximately two weeks after onset of fever, which indicate haemorrhagic pathological alterations developed in vivo [22]. Interestingly, we previously observed VEGFC up-regulation in swine umbilical vein endothelial cells infected with CSFV Shimen [23]. The VEGF family is known to play a central role in the breakdown of clots, angiogenesis, and increased vascular permeability [24]. Our data support the involvement of the VEGF family in the pathological mechanisms of acute CSF and its contribution to haemorrhagic lesions caused by virulent strains of CSFV.

The present study indicated that *PLAU* expression was significantly up-regulated in macrophages infected by CSFV Shimen. uPA plays a vital role in maintaining capillary integrity and regulating vascular permeability and participates in many important processes, including inflammation [7]. A previous study reported uPA up-regulation in HIV-infected macrophages; thus, the protein could aid viral fusion or be an endogenous component critical to HIV infection of macrophages [25]. Additionally, uPA expression in macrophages is believed to contribute to cerebral injury in patients with HIV [26]. Generally, when tissues are challenged by pathogens, macrophages are activated and produce large numbers of pro-inflammatory mediators that kill invading organisms and activate adaptive immunity [27]. However, consistently with previous studies, we believe that infection of macrophages by virulent strains of CSFV directly produces a pro-inflammatory environment leading to prolonged capillary inflammation and vasculitis.

VEGF, which is up-regulated by CSFV Shimen infection, is reportedly the prime factor in the initiation of angiogenesis to increase vascular permeability. Additionally, VEGF reportedly induces uPA activation on the surfaces of endothelial cells to provide further angiogenesis-modulating stimuli [28]. The uPA receptor (uPAR) may be a vehicle by which crucial VEGFA-induced proteolytic factors increase vascular permeability [29]. Consistently with this, VEGF led to the redistribution of uPAR/integrin α5β1 complexes to focal adhesions on the surfaces of endothelial cells, thereby enabling matrix degradation and cell invasion [30]. Furthermore, active uPA increases vascular permeability by increasing VE-cadherin degradation [31]. CSFV may produce its characteristic tissue lesions by targeting uPAR and integrin interaction sites independently of VEGF. Consistently, integrin β3 has is also up-regulated by CSFV infection in host cell lines [32].

## Conclusions

In conclusion, we showed that *VEGFA* and *PLAU* were significantly up-regulated after CSFV Shimen infection. Our results suggest a mechanism of macrophage-mediated inflammatory response to virulent CSFV infections that may explain the as yet indeterminate cause of diffuse haemorrhage in CSF, a highly contagious disease of commercial livestock with a high mortality rate.

## Additional files

Additional file 1: Co-expression network of CSFV Shimen-infected macrophages. (JPG 2474 kb)

Additional file 2: Datasets used to generate co-expression network. (XLS 32 kb)

## Abbreviations

CSF: Classical swine fever
CSFV: Classical swine fever virus
DGE: Digital gene expression
MOI: Multiplicity of infection
PLAU: Plasminogen activator, urokinase
uPA: urokinase-type plasminogen activator
uPAR: uPA receptor
VEGFA: Vascular endothelial growth factor A
