# Supplementary figures and images for: Coordinated expression of vascular endothelial growth factor A and urokinase-type plasminogen activator contributes to classical swine fever virus Shimen infection in macrophages
Source: BMC Vet Res. 2019 Mar 8;15:82. doi: 10.1186/s12917-019-1826-8 (PMC6407193; doi:10.1186/s12917-019-1826-8)

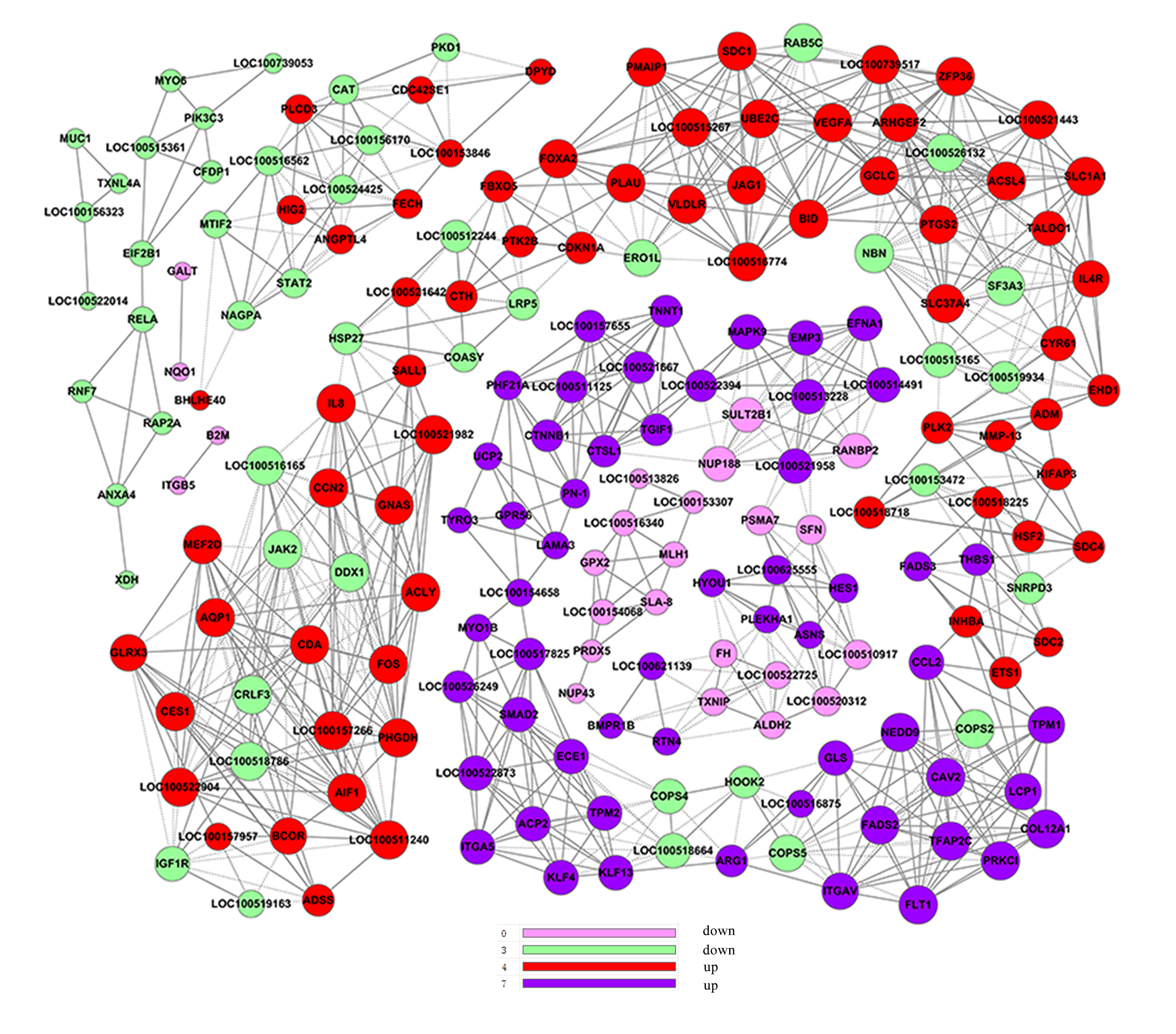

Supplement: Supplementary file 1 — Co-expression network of CSFV Shimen-infected macrophages. (JPG 2474 kb) [file 12917_2019_1826_MOESM1_ESM.jpg]
